# Role of South African Community Pharmacists in Wound Care: An Exploratory Study

**DOI:** 10.3390/ijerph23040470

**Published:** 2026-04-07

**Authors:** Ilse Truter, Janet Barry, Lara Cunningham, Alicia de Lange, Tifany Floors, Donnay Fourie, Sithembile Gumbi, Felicia Lategan, Mohale Leselo, Phelelani Mazibuko, Lukhanyo Ngalo, Sikelela Pangomso, Lisa-Nicole Scholtz, Zanele Tose, Johan Hugo

**Affiliations:** 1 Department of Pharmacy, Nelson Mandela University, P.O. Box 77000, Port Elizabeth (Gqeberha) 6031, South Africa; jbarry@mandela.ac.za (J.B.); lara.cunningham17@gmail.com (L.C.); aliciadelange99@gmail.com (A.d.L.); 130424tifany@gmail.com (T.F.); donnayfourie2@gmail.com (D.F.); cthembiley04@gmail.com (S.G.); lateganfelicia@gmail.com (F.L.); leselomohale@gmail.com (M.L.); mazibukonzima9@gmail.com (P.M.); ngalolukhanyo@gmail.com (L.N.); spangomso83@gmail.com (S.P.); scholtzln@gmail.com (L.-N.S.); tosezanele@gmail.com (Z.T.); 2 Drug Utilization Research Unit (DURU), Nelson Mandela University, P.O. Box 77000, Port Elizabeth (Gqeberha) 6031, South Africa; 3 Department of Statistics, Nelson Mandela University, P.O. Box 77000, Port Elizabeth (Gqeberha) 6031, South Africa; johan.hugo@mandela.ac.za

**Keywords:** diabetic foot, pharmacists, education, health services, wound care

## Abstract

**Highlights:**

**Public health relevance—How does this work relate to a public health issue?**

- Acute and chronic wounds can lead to significant emotional and financial burdens for patients and governments.
- Pharmacists are trained to manage and counsel patients on basic wound care in their undergraduate pharmacy training, with a specific focus on wounds observed in primary healthcare settings.

**Public health significance—Why is this work of significance to public health?**

- Community pharmacists can assist in alleviating the burden that untreated or incorrectly treated wounds may cause.
- Pharmacists and nurses, working together as part of the healthcare team, have complementary skills in wound care that could be used to the benefit of patients.

**Public health implications—What are the key implications or messages for practitioners, policy makers and/or researchers in public health?**

- The role of community pharmacists in wound care in South Africa could be expanded.
- Focus should be placed on better equipping pharmacists with the knowledge necessary to assist patients with wound care management and education.

**Abstract:**

Community pharmacists are in a perfect position to offer comprehensive wound management advice to patients with both chronic and acute wounds, which can result in major costs and emotional burdens. A wound that is managed well through correct assessment, suitable treatment and follow-up counselling will heal optimally and essentially save patients costs and stress. The study aimed to explore the role of South African community pharmacists in wound management. A list of community pharmacies was requested from the South African Pharmacy Council and an online questionnaire was conducted in August 2022 using QuestionPro^®^ Version 2. Stratified random sampling was used to select 350 community pharmacies out of a total of 3240 in South Africa. The response rate was 16.0% (n = 56). Half of the community pharmacists (n = 28) had adequate knowledge about acute wound care, with a third (30.4%, n = 17) indicating that they provide patients with chronic diabetic wound care advice at least once a week. Respondents indicated Continuous Professional Development (CPD) activities and reading journal articles, many relating to diabetic wound management, as ways in which they upskill themselves in wound care. Many of the respondent pharmacists were in favour of an increased focus on wound care services in their community pharmacies. Further training programmes and workshops could be offered to provide pharmacists with the knowledge necessary to manage both chronic and acute wounds in their pharmacies.

## 1. Introduction

### 1.1. Importance of Wound Care

Wounds heal well under ideal circumstances and when they are treated correctly, but there are many factors that can affect the wound healing process. Infection, diabetes, obesity, age, stress, medication, and smoking are just a few examples where wounds may take longer than normal to heal [1]. In these cases, specialised care from a healthcare provider is necessary to advise on the correct treatment regime [2]. The best way to prevent and treat wounds is through an interprofessional team approach, with doctors, pharmacists, and nurses all caring for the patient within their respective scopes of practice and according to the individual patient’s needs [3].

Chronic wounds pose a large burden on healthcare financial resources, and if wounds are not treated correctly, it could result in extended treatment periods and wasting the limited resources available [4]. According to the International Diabetes Federation (IDF), there were over 588 million people living with diabetes globally in 2024, expected to rise to 850 million in 2050 [5]. This chronic disease can lead to conditions that do not heal on their own, such as diabetic foot ulcers, which can become infected. As a result, patients would then need advanced wound care dressings to assist the healing process [6]. Research has shown that the annual National Health Service (NHS) cost in the United Kingdom for wound management (especially diabetic foot ulcers and venous leg ulcers) amounted to more than £8.3 billion in 2017/2018 [7]. Eighty-one per cent of this cost was incurred in the community sector. According to Queen and Harding [8], the estimated wound care spend in 2022 in the United States was $148 billion. In South Africa, the estimated spend on wound care in 2019 was R18.6 billion [9]. Primary healthcare providers treat and manage wounds, since wound care referral is affected by high costs and time [10].

Besides the costs involved in wound care, selecting the correct dressing for a wound can have a huge impact on the healing time. As a result, many factors need to be considered when selecting the correct dressing. These include the type of dressing, the cause and location of the wound, and the availability and cost of the dressings. Understanding all these factors and being able to advise patients on the correct treatment requires an understanding of wound care at the primary health level [1].

Researchers in the United Arab Emirates investigated pharmacists’ understanding of wound causes, healing and management and found that 75% of the participants in the study did not have any specific knowledge on wound management [11]. The majority (78%) of the participants also did not have any reference material in their pharmacies relating to wound management [11]. This lack of knowledge and understanding of a medical condition that costs so much and affects so many patients indicates the potential to train pharmacists to be better able to deal with wound management.

### 1.2. Definition of Advanced Wound Care

Wounds can be classified as either acute or chronic [12]. The main difference between these two classifications is the clarity of the start and endpoint of the wound healing process. Acute wounds have clear start and end stages of healing, while in chronic wounds, the stages of wound healing are not defined [13]. Acute wounds heal in a predictable manner and a relatively short time frame, while chronic wounds require a long time to heal [12]. Research further defines advanced wound care as interventions in wound care when standardised treatment does not work, usually taking longer than 4 to 6 weeks to heal [14]. Common therapeutic treatments for chronic wounds include, but are not limited to, hydrogels, hydrocolloids, alginates and film/foam dressings, antimicrobials, and low-adherent dressings, most of which can be found on the shelves of community pharmacies [15].

### 1.3. The Pharmacist in Wound Care

Pharmacists are often the first point of contact for patients with acute or chronic wounds [1]. Pharmacists are ideally placed in various sectors, such as hospitals and community and primary care, to provide a role in wound management and to facilitate communication between various healthcare practitioners dealing with wounds [1]. Community pharmacies worldwide are no longer just dispensing medicines; they are healthcare providers, offering many services to patients besides being the custodian of medication, including wound care products [16]. Pharmacists’ role has changed from being product-oriented to patient-centred and patients expect a high level of delivery of pharmaceutical services from pharmacists [17].

Treating chronic wounds has moved away from using products that patients readily keep at home to using materials that are manufactured on a large scale and available at a pharmacy or clinic [18]. While pharmacists do want to expand their role in wound management, they face barriers that limit their interactions with patients needing wound care services [19].

Modern advanced wound care products used to treat chronic wounds, such as dressings and skin substitutes, are cost-effective, easy to apply, and readily available [20]. Considering the variation in types of wounds and the number of wound products available, pharmacists do not always possess the knowledge or confidence to provide adequate wound care advice to patients in the pharmacy [1]. In a survey of 92 pharmacists in Dubai, researchers found that 50% of respondents mentioned that they were involved in the management of wounds in their pharmacy, two to three times per week [11]. A qualitative study conducted in Malaysia showed that a lack of wound management knowledge and skills hindered pharmacists’ ability to offer effective advice on wound management and products that could be used to treat wounds. Participants also did not readily want to rate their level of wound care knowledge [19].

In a survey on wound care knowledge in South Africa, researchers said, “Very little, if any, training on chronic wounds is offered in South Africa. However, it is clear that there is a need for improved education about these conditions that have huge clinical and economic consequences” [21]. Additionally, in South Africa, there is a shortage of pharmacists in community settings, which means that they often handle high dispensing volumes and stock control tasks, which limits time for patient-care services such as wound management [22].

In a study done in Australia, pharmacists providing specialised wound care services had attended workshops and also postgraduate wound care training, showing how structured training opportunities can enhance a pharmacist’s ability to provide specialised wound care services [23].

Regulatory bodies encourage the quality of services given by registered pharmacists [17]. Even though this is the case, in South Africa, pharmacists are limited in the services for which they can charge, and counselling on wound care management and products is not included [24]. Pharmacists in community pharmacies are positioned ideally to fill an important role in the management and treatment of wounds by collaborating with the interprofessional team to provide the patient with the best possible care, and with additional knowledge on wound care, they can have an impact on the outcomes [8].

In this study, the role of the community pharmacist in managing advanced wound care was explored. The primary aim of the study was to investigate the role of South African community pharmacists in acute and chronic wound care management. The specific objectives were to:

- Determine which types of wound-related queries pharmacies received most often, and if a statistically significant association existed between wound-related queries received by pharmacies and the area (urban or rural) in which the pharmacies were located.
- Determine descriptively if there is a difference in the types of wounds treated by pharmacies and the specific area (metropolitan (urban) or rural) in which the pharmacies are located.
- Determine the extent of training received by pharmacists on acute and chronic wound care and if an association exists between the type of training received and the specific area in which the pharmacy is located.
- Understand barriers that exist to providing wound care management.

## 2. Materials and Methods

### 2.1. Research Design and Participants

A cross-sectional questionnaire was conducted on a sample of community pharmacies in the private healthcare sector in South Africa. Hospital pharmacies and clinics in the public healthcare sector were excluded.

### 2.2. Data Collection Instrument

A purpose-designed, structured online questionnaire was developed using QuestionPro^®^ Version 2. The questions were based on a thorough literature review on the role of community pharmacists in wound care.

A pilot study was performed after ethical approval for the study had been obtained. For the pilot study, the questionnaire was sent to pharmacists working in other sectors (hospital and academia) to determine how long it would take to complete the questionnaire and also if all the questions were clear and unambiguous. After minor adjustments were made to the questionnaire, the survey was conducted among community pharmacists in South Africa. The structure and questions included in the questionnaire are given in Supplementary Table S1. The questionnaire consisted of six sections. These included demographic information, wound care and training, acute wounds, chronic wounds in general, chronic wounds in diabetic patients, and general comments. The questionnaire was pilot tested and minor amendments were made to enhance clarity.

### 2.3. Sampling

South Africa is divided into nine provinces or regions. A list of all the community pharmacies was requested from the South African Pharmacy Council. From this list, the provincial distribution of the 3240 community pharmacies could be determined (see Table 1). Thereafter, the proportion of pharmacies that needed to be sampled from each province could be determined. Stratified random sampling was used to select 350 pharmacies from a total of 3240 community pharmacies in South Africa. This represented a sample of 10.80% of all community pharmacies in South Africa (last column in Table 1). The list was extracted on 14 June 2022. Only 56 responses were received, representing a response rate of 16.00%.

Corporate (or chain) pharmacies can be defined as pharmacies that are linked to form a group of pharmacies with some central management control (for example, Clicks or Dis-Chem in South Africa). Independent pharmacies can be defined as pharmacies that operate independently of any other pharmacy or group of pharmacies. In the case of corporate pharmacies, a letter was emailed to each regional head office of the corporate pharmacy group (the gatekeepers) to request permission to participate in the study. The names of the pharmacies that belong to each of the corporate pharmacy groups were extracted, and a separate list was compiled for each pharmacy group. Emails with the survey link were sent to each gatekeeper, together with a list of the pharmacies selected from that specific group. Gatekeepers were asked to distribute a letter containing the consent form and questionnaire link to those pharmacies in their corporate group.

In the case of independent community pharmacies, the email with the consent form and link to the survey was emailed directly to the responsible pharmacist of each independent community pharmacy. All pharmacies had to consent before participating in the study. The online questionnaire was conducted during August and September 2022 using QuestionPro^®^. The response rate was 16.0% (n = 56).

### 2.4. Statistical Analysis

The data was analysed using Microsoft Excel^®^ (the latest version that was available as part of Microsoft 365 Apps for Enterprise). For the most part, only basic descriptive statistics (frequencies and percentages) could be determined, since the response rate from rural pharmacies specifically was low (only 10 rural pharmacies responded). However, in order to investigate if significant differences existed between wound care management practices used in pharmacies located in rural or urban areas, it was initially proposed that chi-squarse tests of homogeneity be conducted to compare whether the two groups (rural pharmacies and urban pharmacies) were homogeneous with regard to proportions across different categories of a second categorical variable. In other words, the chi-square tests were conducted to see if there was an association between the area (rural or urban) in which the pharmacy was located and the specific wound care management practices used. Contingency tables therefore had to be constructed. Whilst running the analysis, it was discovered that, since the response rate amongst rural pharmacies was low, the basic assumption pertaining to expected frequencies used when calculating the chi-square test statistic was not met. Specifically, more than 20% of the expected frequencies calculated in each cell of the contingency table were less than 5 [25]. It was therefore decided to instead use Fisher’s Exact Test, since this test is valid for all sample sizes, especially when more than 20% of expected frequencies are less than 5 [25]. The effect size, as measured by Cramer’s V, was also determined and will be reported in cases where the null hypothesis of no association between the two categorical variables was rejected. Reporting effect size using Cramer’s V is relevant in this case, since the contingency tables were larger than 2 × 2 (that is, one categorical variable had more than two levels) [25].

### 2.5. Ethical Considerations

Informed consent was obtained from all participants before taking part in the study. Ethical approval for the study was obtained from the Nelson Mandela University Research Ethics Committee (Human) (registration number: H22-HEA-PHA-004). Generative artificial intelligence (GenAI) has not been used in this paper.

## 3. Results

### 3.1. Demographic Information

Most respondents consenting to take part in the survey (n = 51) were from the corporate community pharmacy sector; only four respondents were from the independent community pharmacy sector, and one respondent did not indicate the sector their pharmacy is located in. Most pharmacies were located in Gauteng (n = 21) and the Western Cape (n = 12) provinces. Table 2 shows the number of pharmacies located in the different provinces according to their respective rural or urban setting. From Table 2, it can be seen that most pharmacies (n = 44) were located in urban (or metropolitan) areas, with only 10 pharmacies located in rural areas. No pharmacies from the Northern Cape province participated in the study.

Most respondents had more than 10 years’ experience as a registered pharmacist working in community pharmacies (45.45%), followed by 3 to 5 years’ experience (29.09%) and 6 to 10 years’ experience (21.82%).

Only half of the pharmacies (50.00%) had a clinic inside their pharmacy where wounds could be assessed and treated. Twenty-seven pharmacies did not have a clinic, with seven of these pharmacies located in rural areas and 20 in urban areas.

Nearly half (46.30%) of the pharmacies employed a nursing sister. Of these pharmacies, 50.00% were located in rural areas, while the remaining 50.00% were located in urban areas.

### 3.2. Wound Care Services and Training

Table 3 shows general information about the wound care services provided in the community pharmacies, as well as training in wound care.

For the results provided in Table 3, using the Fisher Exact Test to test for a significant association between the frequency of advice on wound care provided in the pharmacy and where the pharmacies are located (urban or rural), it was found that a statistically significant association existed between these two categorical variables at the 5% level of significance (*p*-value = 0.01204). As mentioned, the effect size, as measured by Cramer’s V (Cramer’s V = 0.17638, 0.17 < Cramer’s V < 0.29, df = 3), was also determined and indicated a medium-to-large effect size when interpreted [25].

When considering the Fisher Exact Test to test for a significant association between the length of time, on average, that it takes for the pharmacist or registered nurse to perform a wound care service on a patient and the area (rural or urban) the pharmacies are located in, it was found that, at a 1% level of significance, a significant association existed between these two categorical variables (*p*-value = 0.00327). This *p*-value can be considered highly significant. The effect size, using Cramer’s V (Cramer’s V = 0.22318, 0.17 < Cramer’s V < 0.29, df = 3), was also determined, and, similar to the previous case, it represented a medium-to-large effect size [25].

Fisher’s Exact Test was also used to determine if a significant association existed between the manner in which pharmacy staff stay up to date with chronic wound care management and the area (rural or urban) in which the pharmacies were located. The results again indicated that at a 5% level of significance, a statistically significant association existed between these two variables (*p*-value = 0.01325), with Cramer’s V (Cramer’s V = 0.08835, 0.06 < Cramer’s V < 0.17, df = 3), however, only indicating a small-to-medium effect size [25].

Lastly, a Fisher Exact Test was also conducted to determine if a statistically significant association existed between the number of staff in the pharmacy with a current first aid certification and the area (rural or urban) in which the pharmacies were located. The results from the Fisher Exact Test indicated that a highly statistically significant association existed between these two variables (*p*-value = 0.00041) at a 1% level of significance, with Cramer’s V (Cramer’s V = 0.21088, 0.15 < Cramer’s V < 0.25, df = 4) again indicating a medium-to-large effect size.

### 3.3. Acute Wounds

Pharmacists rated their knowledge of acute wound management, from “1 = Very little knowledge” to “6 = Excellent knowledge”. The results are given in Table 4. The different types of acute wounds that are encountered in community pharmacies were listed and respondents were asked to rank them in terms of how often they encounter these wounds in their respective pharmacies. The results are given in Table 5.

Respondents were asked to list any other acute wounds, not mentioned in the options in Table 5, that were observed in the pharmacies where they work. Ten pharmacists listed additional wounds. Among these additional listings were animal and insect bites, and diabetic wounds were also mentioned.

On the question of which acute wounds pharmacists would recommend a vaccination for and which vaccines would be recommended, animal bites and lacerations were the most frequently mentioned (34 pharmacists responded), with the tetanus and rabies vaccines recommended most frequently.

### 3.4. Chronic Wounds in General

Eight types of chronic wounds were listed, namely Venous Ulcers, Arterial Ulcers, Diabetic Foot Ulcers, Pressure Ulcers, Infected Wounds, Ischemic Wounds, Surgical Wounds and Radiation Poisoning Wounds. Infected wounds were mentioned by 64.29% of the respondents, followed by surgical wounds (51.79%) and diabetic foot ulcers (44.64%). Radiation Poisoning Wounds and arterial ulcers were encountered the least. Respondents were asked to list any other chronic wounds that they encounter in their pharmacy. Only cellulitis, which is not an actual wound itself, was mentioned.

**Figure 1:**
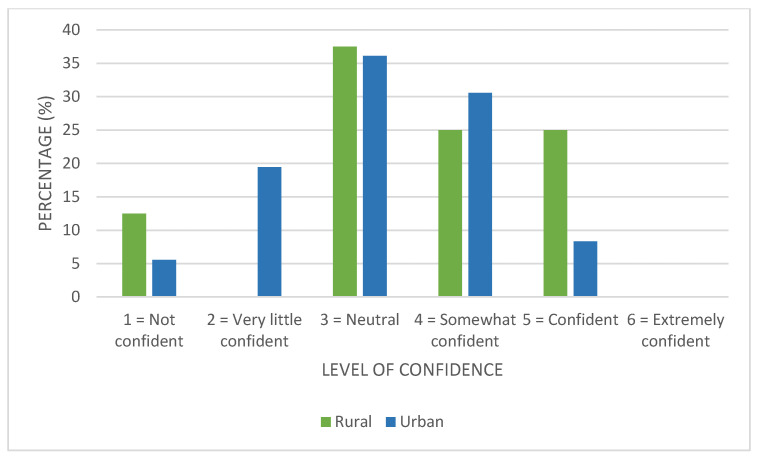
Level of confidence of pharmacists in rural and urban areas in managing chronic wounds (n = 44).

Respondents were also asked to rate how confident they feel when asked to give advice to a patient on how to manage their chronic wounds (1 = Not confident; and 6 = Extremely confident). The results are given in Figure 1 for rural and urban pharmacies. The mode (most frequently occurring selection) in both rural and urban settings was 3, followed by a score of 4. None of the pharmacists indicated that they were “extremely confident”.

**Figure 2:**
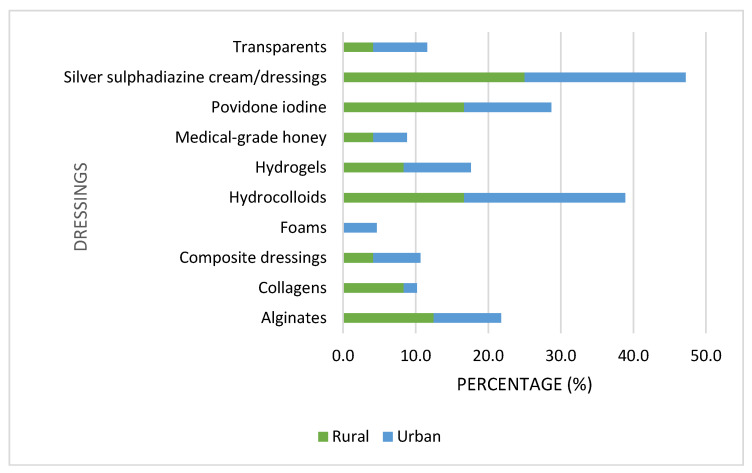
Dressings that are used in managing chronic wounds (n = 132).

Respondents were asked which dressings they recommend for chronic wounds. Dressings that were recommended for chronic wounds are listed in Figure 2. More than one option could be marked (a total of 132 options were marked). Silver sulphadiazine cream/dressings and hydrocolloids were the most popular. Other dressings that were listed as used but not included in the list included gauze. Respondents also mentioned certain ointments and creams. These items, however, are not considered dressings.

### 3.5. Chronic Wounds in Diabetic Patients

Respondents were asked how often they provide pharmaceutical care in the form of educating patients about the risk of diabetic chronic wounds. Forty-four respondents answered this question. Most respondents (69.57%), in both rural and urban areas, indicated less than once a week. Only one respondent indicated more than three times a week.

Respondents were asked which grades, according to the Wagner Grading Scale [26], of diabetic foot ulcers they encounter in the pharmacies where they work. A photo and a brief description of each grade was given. Table 6 illustrates the grades of diabetic foot ulcers that pharmacists have observed in the pharmacies where they work (more than one option could be selected).

## 4. Discussion

Most community pharmacies in South Africa are located in Gauteng, KwaZulu-Natal and the Western Cape, and in urban areas. This study included respondents from 10 rural and 44 urban settings (see Table 1). Community pharmacies in urban areas are generally associated with higher population density and stronger economic activity. In contrast, rural areas typically have smaller populations and lower levels of economic activity [27]. Since it was shown in this study that statistically significant associations existed between the wound care services provided and the area (rural or urban) in which the pharmacies were located, this may have implications for the availability of wound care services in rural areas, which often constitute farming communities and occupations in the informal sector, where wounds may be more prevalent. A lack of wound care services in these areas may therefore have negative effects on the health of the population. The Northern Cape Province, the least populous province in the country, with only 63 community pharmacies at the time of the study, as mentioned, did not respond in this study.

Half of the pharmacies in this study (n = 28) indicated that they had a clinic inside their pharmacy where wounds could be assessed and treated. A clinic is usually referred to in legislation as an isolated consulting area that is used for screening and monitoring services. According to Good Pharmacy Practice [28], the pharmacist may use this area to consult with a patient in private and/or perform various services (for example, cholesterol tests, blood pressure monitoring and pregnancy tests). The clinic is also the area where wound care services can be provided by the pharmacist. In some pharmacies, a nurse is employed, and in such instances, the pharmacy will also have a separate clinic area from which the nurse can provide primary healthcare services.

Acute wounds commonly seen in community pharmacies were abrasions (n = 34), burn wounds (n = 53), surgical incisions (n = 52) and lacerations (n = 48). These acute wounds are very commonly seen in pharmacies, and many of them may need immediate treatment, necessitating the availability of healthcare services. Similar to Nyamuzihwa and others [27] who indicated that community pharmacies present a unique opportunity to help South Africa alleviate the burden on public health systems and overcome the challenges in HIV testing and treatment in South Africa, the authors are of the opinion that community pharmacies can also play a bigger role in wound care services and especially wound prevention in communities, with specific reference to diabetic foot ulcers.

The role of community pharmacy in wound care is varied and includes wound-related and non-wound-specific clinical and non-clinical activities [29]. Wound care management and the dressing of wounds represent some of the most basic services offered in primary healthcare clinics across South Africa, yet in a study conducted in South Africa in 2010, it was found that undergraduate students who left medical school were not equipped with the necessary knowledge to treat chronic wounds [30].

Even though these activities are within the scope of entry-level competency for pharmacists, studies have highlighted the need for additional training when it comes to chronic wounds such as diabetic foot ulcer management [31] A study conducted in South Africa [32] reported on the high prevalence rates of hard-to-heal wounds and delayed referral in the heterogenous South African population and stated that their findings necessitate recognition of wound management as a specialty.

When considering the aforementioned information, there is no current estimate in the literature for the number of people affected in South Africa or the associated costs involved, with very few international studies roughly estimating similar types of numbers or costs. In this study, pharmacists also indicated that they have a sufficient level of confidence in treating chronic wounds.

With respect to chronic wound management, the most common chronic wounds encountered in community pharmacies were infected wounds, surgical wounds and diabetic foot ulcers. This result is consistent with the literature, which reported that 10% to 15% of all patients with diabetes mellitus will acquire a foot infection at some stage in their lifetime [33]. Respondents were of the opinion that they had a very similar understanding of chronic wound management to that of acute wounds, with 65% (n = 46) indicating between 3 (some knowledge) and 4 (adequate knowledge) on the scale. It was noted that there were eight fewer respondents who answered the question about chronic wounds compared to acute wounds, and this could account for the similarity with acute wound understanding.

Pharmacists’ confidence in treating wounds indicated that they are confident in treating acute and less serious wounds. However, with more complicated and chronic wounds, they feel less confident. Wound care in the undergraduate pharmacy curriculum in South Africa focuses primarily on wounds commonly seen in primary healthcare and not so much on the treatment of chronic and more extensive wounds. The possibility of further training of pharmacists in wound care exists, where the focus can be on the identification and grading of wounds, the use of the different wound care products on the market, and also, especially, the continuous monitoring (and referral where required) of patients with chronic wounds. Upskilling pharmacists in wound care can either be achieved by involvement in Continuous Professional Development (CPD) activities related to chronic wound management, as was indicated by half (n = 28) of the respondents, or by educating themselves by reading up on new developments in chronic wound care management (n = 27). Ideally, wound care training should be hands-on and in-person, so that pharmacists can observe the various types and degrees of wounds and familiarise themselves with the application of the various wound care products on the market.

Limitations of the study included the relatively low response rate of only 16% and the fact that corporate pharmacies were overrepresented in this study. Also, no pharmacies located in the Northern Cape province participated in the study. This unfortunately represented a largely rural province with large farming communities and relatively sparse healthcare services. Respondents also responded based on how they perceive their knowledge of wound management (self-reporting) and there may also have been selection bias. Despite these limitations, the study provides a broad overview of the current role that community pharmacists in the private healthcare sector play in wound care. Further studies are recommended on wound care services and can also include wound care services in the public healthcare sector in South Africa to provide a more comprehensive overview of wound care in the country.

## 5. Conclusions

Community pharmacists in the private sector of South Africa currently play an important role in acute and chronic wound care services, which in future may be even more important. Greater focus should therefore be placed on training programmes and workshops to better equip pharmacists with the knowledge necessary to assist patients with wound care management and education, especially considering the areas in which the pharmacies are located. The study highlighted a keen interest from pharmacists for wound care management, from a desire to improve their skills to finding solutions to overcome the challenges they face when providing wound care to patients. Further studies are recommended to identify ways in which wound care management can become a service for which pharmacists may levy a fee (similar to, for example, blood pressure and blood glucose monitoring, the administration of injections, reproductive healthcare services, and various other services). This will enable pharmacists to be remunerated for spending the time counselling patients with wounds, as well as for services rendered, such as cleaning and dressing wounds.

## Figures and Tables

**Table 1:** Provincial distribution of community pharmacies in South Africa on 14 June 2022 with the number of pharmacies sampled from each stratum also indicated.

Province	Community Pharmacies		Number of Pharmacies
	Number	Percentage (%)	Sampled from Each Stratum
Eastern Cape	285	9	31
Free State	151	5	16
Gauteng	1088	34	118
KwaZulu-Natal	540	17	58
Limpopo	196	6	21
Mpumalanga	241	7	26
North West	191	6	21
Northern Cape	63	2	7
Western Cape	485	15	52
Total	3240	100	350

**Table 2:** Number of community pharmacies according to their rural or urban setting consenting to complete the survey (n = 54).

Provinces	Rural (n = 10)	Urban (n = 44)	Total	
			Number *	Percentage (%)
Eastern Cape	0.00	13.64	6	11.11
Free State	10.00	2.27	2	3.70
Gauteng	20.00	43.18	21	38.89
KwaZulu-Natal	20.00	4.55	4	7.41
Limpopo	10.00	2.27	2	3.70
Mpumalanga	30.00	6.82	6	11.11
North West	0.00	2.27	1	1.85
Western Cape	10.00	25.00	12	22.22
Total	100.00	100.00	54	100.00

* Two participants did not indicate if their respective settings were rural or urban.

**Table 3:** Wound care services in community pharmacies.

Wound Care Services	Rural Area % (n)	Urban Area % (n)	Total % (n)
Frequency of advice on wound care provided in the pharmacy (e.g., counselling a patient on how to treat a wound without actually treating the wound)			
At least once or twice a week	20.00 (2)	36.36 (16)	33.33 (18)
Never	0 (0)	2.27 (1)	1.85 (1)
On a daily basis	40.00 (4)	11.36 (5)	16.67 (9)
Once in a while	40.00 (4)	50.00 (22)	48.15 (26)
Total	100.00 (10)	100.00 (44)	100.00 (54)
Length, on average, that it takes for the pharmacist or registered nurse to perform a wound care service on a patient			
15–24 min	10.00 (1)	11.36 (5)	11.11 (6)
25 min or longer	10.00 (1)	18.18 (8)	16.67 (9)
5–14 min	40.00 (4)	59.09 (26)	55.56 (30)
Less than 5 min	40.00 (4)	6.82 (3)	12.96 (7)
Not answered	0 (0)	4.55 (2)	3.70 (2)
Total	100.00 (1)	100.00 (44)	100.00 (54)
How pharmacy staff stay up to date with chronic wound management			
Pharmacists educate themselves by reading up on new developments in chronic wound care management	3	24	27
Pharmacists do Continuous Professional Development (CPD) * activities related to chronic wound care management	7	21	28
Pharmacists attend chronic wound care workshops/seminars/webinars in order to learn about the newest products on the market	1	6	7
Pharmacists rarely attend workshops or do their own research on chronic wound care management	3	14	17
Number of staff in the pharmacy with current first aid certification			
None	30.00 (3)	7.14 (3)	11.54 (6)
One	50.00 (5)	33.33 (14)	36.54 (19)
Two	0 (0)	30.95 (13)	25.00 (13)
Three	20.00 (2)	14.29 (6)	15.38 (8)
Four or more	0 (0)	14.29 (6)	11.54 (6)
Total	100.00 (10)	100.00 (42)	100.00 (52)

* Continuous Professional Development (CPD) is the process by which registered pharmacy staff maintain and enhance their competence throughout their careers. It encompasses learning activities (including education and training) to safeguard the public and uphold professional integrity.

**Table 4:** Knowledge of acute wound management (n = 53).

Rating of Their Own Knowledge of Acute Wound Management	Rural Area % (n)	Urban Area % (n)	Total % (n)
1—Very little knowledge	0 (0)	4.65 (2)	3.77 (2)
2—Little knowledge	10.00 (1)	11.63 (5)	11.32 (6)
3—Knowledge; however, not adequate	20.00 (2)	39.53 (17)	35.85 (19)
4—Some adequate knowledge	40.00 (4)	34.88 (15)	35.85 (19)
5—Good knowledge	20.00 (2)	9.30 (4)	11.32 (6)
6—Excellent knowledge	10.00 (1)	0 (0)	1.89 (1)
Total	100.00 (10)	100.00 (43)	100.00 (53)

**Table 5:** Frequency of the different acute wounds encountered in community pharmacies.

Types of Acute Wounds	Rural Area % (n)	Urban Area % (n)	Total % (n)
Surgical incisions			
1—Most commonly observed	10.00 (1)	26.19 (11)	23.08 (12)
2—Sometimes observed	30.00 (3)	14.29 (6)	17.31 (9)
3—Seldomly observed	10.00 (1)	23.81 (10)	21.15 (11)
4—Least commonly observed	50.00 (5)	35.71 (15)	38.46 (20)
Total	100.00 (10)	100.00 (42)	100.00 (52)
Burn wounds			
1—Most commonly observed	40.00 (4)	32.56 (14)	33.96 (18)
2—Sometimes observed	0 (0)	32.56 (14)	26.42 (14)
3—Seldomly observed	50.00 (5)	20.93 (9)	26.42 (14)
4—Least commonly observed	10.00 (1)	13.95 (6)	13.21 (7)
Total	100.00 (10)	100.00 (43)	100.00 (53)
Abrasions			
1—Most commonly observed	40.00 (4)	25.58 (11)	28.30 (15)
2—Sometimes observed	10.00 (1)	34.88 (15)	30.19 (16)
3—Seldomly observed	40.00 (4)	25.58 (11)	28.30 (15)
4—Least commonly observed	10.00 (1)	13.95 (6)	13.21 (7)
Total	100.00 (10)	100.00 (43)	100.00 (53)
Lacerations			
1—Most commonly observed	10.00 (1)	15.79 (6)	14.58 (7)
2—Sometimes observed	60.00 (6)	18.42 (7)	27.08 (13)
3—Seldomly observed	0 (0)	34.21 (13)	27.08 (13)
4—Least commonly observed	30.00 (3)	31.58 (12)	31.25 (15)
Total	100.00 (10)	100.00 (38)	100.00 (48)

**Table 6:** Grades of diabetic foot ulcers seen in pharmacies.

Grades of Diabetic Foot Ulcers	Rural	Urban	Total
Grade 0 This foot has no open lesions, but one may see hammertoes, prominent metatarsals, or other deformities that could lead to pressure-induced ulcerations	3	24	27
Not answered	7	20	27
Total	10	44	54
Grade 1 This foot presents with superficial ulceration through the skin only—no significant wound depth and no evidence of penetrating infection	2	14	16
Not answered	8	30	38
Total	10	44	54
Grade 2 This type of lesion is deeper and may slough down to the tendon, ligament or joint	1	11	12
Not answered	9	33	42
Total	10	44	54
Grade 3 Deep infection progresses to abscess or osteomyelitis. Such a wound requires incision, drainage and debridement of all infected soft tissue or bone	0	2	2
Not answered	10	42	52
Total	10	44	54
Grade 4 Gangrene affects some portion of the forefoot—some type of amputation will be necessary; this may be limited to the toes	0	1	1
Not answered	10	43	53
Total	10	44	54

## Data Availability

The dataset used and analysed during the study is not publicly available because it contains information that could compromise the privacy of research participants, but de-identified datasets are available from the corresponding author on reasonable request.

## Supplementary Materials

The following supporting information can be downloaded at: https://www.mdpi.com/article/10.3390/ijerph23040470/s1, Table S1. Questionnaire Structure and Questions.

## Abbreviations

The following abbreviations are used in this manuscript:

COVID-19	Coronavirus disease 2019
CPD	Continuous Professional Development
WHO	World Health Organization
