# Supplementary material for: Role of South African Community Pharmacists in Wound Care: An Exploratory Study
Source: Int J Environ Res Public Health. 2026 Apr 7;23(4):470. doi: 10.3390/ijerph23040470 (PMC13116394; doi:10.3390/ijerph23040470)
Supplement: Supplementary file 1 [file ijerph-23-00470-s001.zip › ijerph-4204132-supplementary.pdf]

International Journal of
Environmental Research and Public Health

MDPI

SUPPLEMENTARY

Table S1. Questionnaire Structure and Questions

SECTION A: DEMOGRAPHIC INFORMATION	SECTION B: WOUND CARE AND TRAINING	SECTION C: ACUTE WOUNDS	SECTION D: CHRONIC WOUNDS IN GENERAL	SECTION E: CHRONIC WOUNDS IN DIABETIC PATIENTS	SECTION F: GENERAL
In which province is your pharmacy located?	How often is advice on wound care provided in your pharmacy?	Identify the most common acute wounds in your pharmacy.	Identify the most common chronic wounds in your pharmacy.	How often do you provide pharmaceutical care in the form of educating patients about the risk of diabetic chronic wounds?	Describe in a few sentences what are the challenges pharmacy staff face when providing wound care to patients.
Where is your pharmacy located? (Urban, Rural.)	How long, on average, does it take to do a wound care assessment on a patient?	For which acute wounds would you recommend a vaccination, and name the vaccines you recommend?	Which of the following wound care dressings and topical products do you sell in the pharmacy to assist chronic wound patients with managing their wounds?	On a scale of 1 to 5, indicate how confident you are to educate a patient on diabetic wound care.	Are you in support of an increased focus on wound care services in community pharmacies? Please motivate your answer.
Is your pharmacy a corporate or independent pharmacy?	How many staff in your pharmacy have first aid training?		On a scale from 1 to 7, rate how confident you will feel when asked to give advice to a patient on how to manage their chronic wounds.	Indicate which of the following dressings you recommend for chronic diabetic wounds, and whether your pharmacy keep stock.	Please feel free to add any other comments that you think would be useful while exploring this topic?

<table>
<tr><td>How long have you been a registered pharmacist working in community pharmacy?</td><td rowspan="3">Please choose the options applicable to the pharmacy staff with respect to staying up to date with chronic wound care management:
We do our own research on new developments in chronic wound care management.
We do CPD activities related to chronic wound care management.
We attend chronic wound care workshops/seminars/webinars in order to learn about the newest products on the market.
We rarely attend workshops or do our own research on chronic wound care management.</td><td></td><td>On a scale from 1 to 5, rate how confident you will feel when asked to give advice to a patient on how to manage their chronic venous ulcer.</td><td>Indicate from most frequently seen grade/stage of diabetic foot ulcers recurring in your pharmacy to least common.</td><td></td></tr>
<tr><td>Please indicate how many (if any) of the following staff work in your community pharmacy? (Pharmacist, Post Basic Pharmacist, Assistant Basic Pharmacist, Assistant Nursing Sister.)</td><td></td><td></td><td></td><td></td></tr>
<tr><td>Do you have a clinic in the pharmacy where wounds are treated and assessed?</td><td></td><td></td><td></td><td></td></tr>
</table>

Disclaimer/Publisher's Note: The statements, opinions and data contained in all publications are solely those of the individual author(s) and contributor(s) and not of MDPI and/or the editor(s). MDPI and/or the editor(s) disclaim responsibility for any injury to people or property resulting from any ideas, methods, instructions or products referred to in the content.

https://doi.org/10.3390/ijerph23040470
